# Autophagy deficiency promotes M1 macrophage polarization to exacerbate acute liver injury via ATG5 repression during aging

**DOI:** 10.1038/s41420-021-00797-2

**Published:** 2021-12-20

**Authors:** Rui Liu, Juanjuan Cui, Yating Sun, Wentao Xu, Ziming Wang, Miaomiao Wu, Huke Dong, Congcong Yang, Shaocheng Hong, Shi Yin, Hua Wang

**Affiliations:** 1 Department of Oncology, the First Affiliated Hospital of Anhui Medical University, Hefei, 230022 China; 2 Inflammation and Immune Mediated Diseases Laboratory of Anhui Province, Anhui Medical University, Hefei, 230032 China; 3 Department of Stomatology, the First Affiliated Hospital of Anhui Medical University, Hefei, 230022 China; 4 Department of Genetics, School of Life Science, Anhui Medical University, Hefei, 230032 China; 5 First Clinical Medical College of Anhui Medical University, Hefei, 230036 China; 6 Department of Geriatrics, the First Affiliated Hospital of USTC, Division of Life Sciences and Medicine, University of Science and Technology of China, Hefei, Anhui 230001 China

**Keywords:** Senescence, Macroautophagy, Monocytes and macrophages, Inflammation, Hepatotoxicity

## Abstract

Aging disrupts the maintenance of liver homeostasis, which impairs hepatocyte regeneration and aggravates acute liver injury (ALI), ultimately leading to the development of acute liver failure (ALF), a systemic inflammatory response, and even death. Macrophages influence the progression and outcome of ALI through the innate immune system. However, it is still unclear how macrophages regulate ALI during aging. The variation in macrophage autophagy with aging and the influence on macrophage polarization and cytokine release were assessed in BMDMs in vitro. Then, after BMDMs subjected to several treatments were intravenously or intraperitoneally injected into mice, thioacetamide (TAA)-induced ALI (TAA-ALI) was established, and its effects on inflammation, injury, and mortality were assessed. We found that aging aggravated the liver injury, along with increases in the levels of proinflammatory mediators, presenting a senescence-associated secretory phenotype (SASP), which promoted macrophage polarization to the M1 phenotype. In addition, autophagy levels decreased significantly in aged mice, which was ascribed to ATG5 repression during aging. Notably, enhancing autophagy levels in aged BMDMs restored macrophage polarization to that observed under young conditions. Finally, autophagy restoration in aged BMDMs enhanced the protective effect against TAA-ALI, similar to M2 macrophages induced by IL-4. Overall, we demonstrated that the influence of aging on macrophage polarization is an important aggravating factor in TAA-ALI, and the autophagy in macrophages is associated with the aging phenotype.

## Introduction

As the main site of nutrient and toxin metabolism and a barrier to complex pathogenic factors from the external environment [1], the liver can be damaged by multiple toxins and their metabolites, but it usually regenerates after moderate injury due to its remarkable regeneration capacity [2]. However, liver regeneration may fail after some severe acute insults, leading to acute liver injury (ALI) and even acute liver failure (ALF). ALF is a rare and severe consequence of intense liver damage that can cause death within days or weeks [3]. Clinically, ALI is more likely to progress to ALF in elderly individuals, who have a poorer prognosis and higher mortality than young individuals [4]. Therefore, ALF has important clinical significance in previously healthy individuals, but it is still unclear why ALI is more inclined to progress to ALF as a result of aging.

Appropriate inflammation helps liver repair, but excessive inflammation will further promote hepatocyte injury and death [5]. When hepatic inflammation is out of control, innate immune cells (macrophages, neutrophils, dendritic cells, etc.) recruit and release cytokines, chemokines, and reactive oxygen species, thereby inducing hepatocyte apoptosis and necrosis [6]. In turn, dying hepatocytes release damage-associated molecular patterns (DAMPs), which bind to evolutionarily conserved pattern recognition receptors, activate the innate immune system, and stimulate inflammatory responses, ultimately creating a vicious cycle of severe damage to hepatocytes [7]. Body senescence is accompanied by organ senescence and cellular senescence (including immune cell senescence). Senescent immune cells develop immunoregulation disorders, expressing numerous senescence-associated secretory phenotypes (SASPs) and secreting various proinflammatory mediators [8]. Thus, understanding the mechanisms by which the senescent liver loses its immunologic balance is essential to delay immune disorders and prevent poor outcomes.

Macrophages are most abundant in the liver among all solid organs in the body, with 20–40 macrophages for every 100 hepatocytes in the rodent liver [9], and they play a crucial role in maintaining hepatic homeostasis [6]. Macrophages can usually differentiate into the M1 phenotype under lipopolysaccharide (LPS)/IFN-γ stimulation, showing a proinflammatory and injury-promoting phenotype, or into the M2 phenotype under IL-4 stimulation, showing an anti-inflammatory and tissue-protective phenotype [10, 11]. Studies have shown that the ratio of M1 to M2 macrophages changes under pathological conditions during aging [12, 13]. In addition, in aged healthy adults, macrophages in fat and liver tissues showed a more proinflammatory M1 phenotype, while the immunosuppressive M2 phenotype was increased in the lymphoid tissues, lungs, and muscles [10]. Exploring aging-related polarization mechanisms is crucial to recognize the role of macrophages in ALI, and interventions targeting macrophage polarization may be used to achieve favorable outcomes.

Macroautophagy (hereafter referred to as autophagy) is an adaptive and protective mechanism that controls cellular stress, protein homeostasis, and mitochondrial quality [14]. Recent evidence suggests that autophagy in macrophages can help reduce inflammation and injury induced by D-galactosamine (D-gal)/LPS [15], carbon tetrachloride (CCl_4_) [16], and nonalcoholic steatohepatitis (NASH) [17]. ATG genes control the formation of autophagosomes through ATG12-ATG5 and LC3-II complexes. Multiple studies have shown that ATG5 plays an essential role in autophagy regulation. Lodder et al. reported that liver fibrosis in ATG5 knockout mice showed higher IL-1α and IL-1β levels and remarkable recruitment of macrophages and neutrophils, which are associated with aggravated liver injury [18]. In D-gal/LPS-induced liver failure, loss of ATG5 significantly increased IL-1β expression, with massive infiltration of neutrophils and aggravated liver failure [15]. Thus, we hypothesized that altered macrophage autophagy regulated by ATG5 plays a crucial role in aging-dependent ALI aggravation.

Thioacetamide (TAA), which is metabolized to its S-oxide (TASO) and further to S, S-dioxide (TASO_2_) through the biological oxidation of cytochrome P450 (CYP) 2E1 in vivo [19], is a classic hepatotoxic agent that induces acute or chronic liver inflammation, liver injury and even liver failure, which is associated with oxidative stress, centrilobular necrosis and apoptosis [20, 21]. In this study, we investigated how autophagy affects macrophage polarization during aging and presented the roles and mechanisms of this process in TAA-ALI.

## Results

### Aging exacerbates TAA-induced ALI

**Fig. 1:**
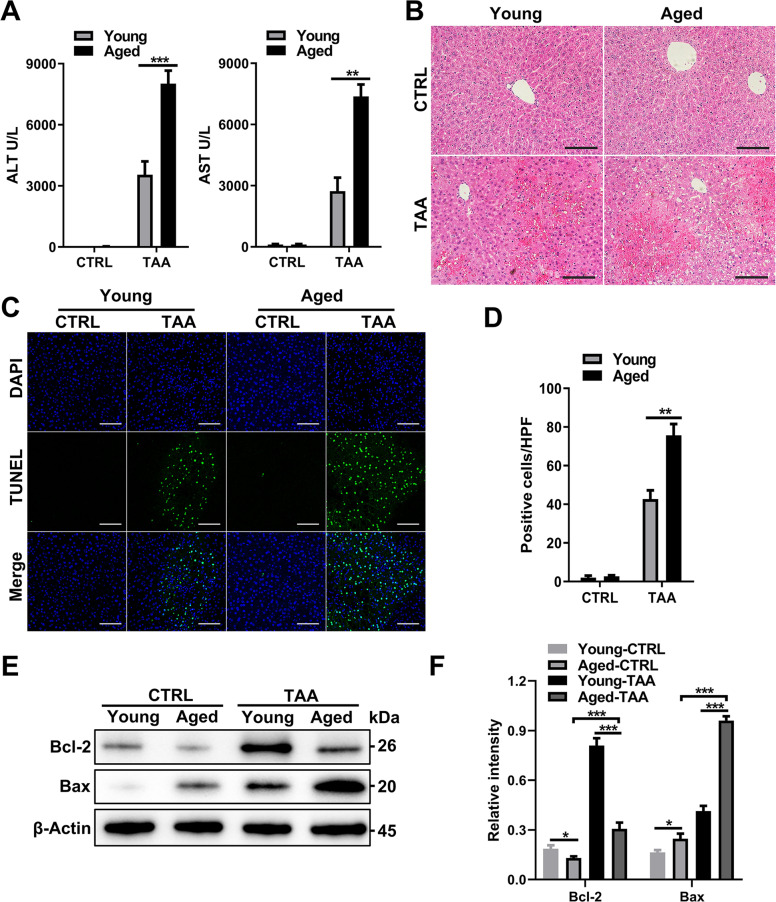
Aging exacerbates liver injury and inflammation after TAA treatment. Young and aged mice were administered TAA (200 mg/kg) and PBS as a control. **A** Serum ALT (left panel) and AST (right panel) levels in each group. **B** Representative H&E staining of the liver. **C** Representative TUNEL (green fluorescence) staining of the liver with DAPI counterstaining (blue fluorescence) and **D** quantification. **E** Immunoblotting of Bax and Bcl-2 expression in liver tissue from each group and **F** quantification. All data shown represent *n* = 8–10 mice per group. All results are representative of at least three independent experiments. Values are presented as the mean ± SD. *p ≤ 0.05; **p ≤ 0.01; ***p ≤ 0.001.

TAA induces increased hepatocyte apoptosis and necrosis. We administered TAA to mice to establish a TAA-ALI model in young and aged mice. The aged mice were less viable and showed reduced consciousness after TAA treatment, and their serum ALT and AST levels were significantly higher than those of young mice (Fig. 1A). Consistent with the serum indexes, H&E staining of liver sections showed more necrotic hepatocytes in aged mice, and the hepatic lobule structure was blurred, indicating that aged mice have weakened tolerance to toxins (Fig. 1B). TUNEL staining showed that the livers of aged mice were more prone to apoptosis and necrosis (Fig. 1C, D). We further detected the expression of the apoptosis-related proteins Bcl-2 and Bax. The expression of the antiapoptotic factor Bcl-2 decreased in aged mice, while the expression of the apoptotic protein Bax increased (Fig. 1E, F). These results indicated that aging aggravates hepatocyte apoptosis and necrosis, thus reducing tolerance to TAA-ALI.

### Aging aggravates inflammation in TAA-ALI

**Fig. 2:**
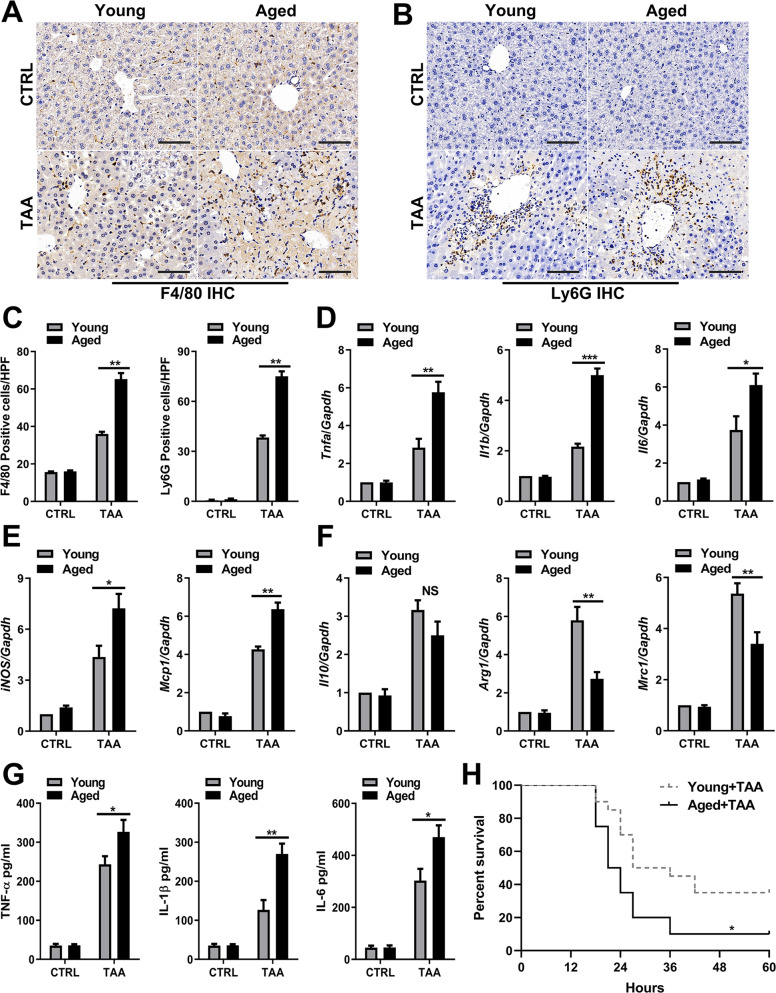
Aging aggravates the inflammatory response in TAA-ALI. Young and aged mice were administered TAA (200 mg/kg) and PBS as a control. **A** Representative images of F4/80 IHC staining of liver tissue from young and aged mice with TAA-ALI. **B** Representative images of Ly6G IHC staining of liver tissue from young and aged mice with TAA-ALI. **C** Quantification of A (left) and B (right). **D**–**F** mRNA expression (*Tnfa, Il1b, Il6, Il10, Arg1, iNOS, Mrc1*, and *Mcp1*) in liver tissue was detected by RT–PCR. The average target gene/GAPDH ratios of different experimental groups relative to the control group are given. (**G**) Determination of the total protein content (TNF-α, IL-1β, and IL-6) in the serum by ELISA. (**H**) Summary of mouse mortality in each group. A high TAA dose of 500 mg/kg was used to calculate mortality and 20 mice per group were involved in mortality analysis. Quantification is shown in the right panel. All results are representative of at least three independent experiments. Values are presented as the mean ± SD. *p ≤ 0.05; **p ≤ 0.01; ***p ≤ 0.001.

To further investigate hepatic inflammation in young and aged mice during TAA-ALI, immunohistochemistry (IHC) and mRNA expression analysis of the liver were performed. IHC assays showed increased macrophage (Fig. 2A, C) and neutrophil (Fig. 2B, C) infiltration in the liver in aged mice during TAA-ALI, indicating increased recruitment of innate immune cells. Examination of liver mRNA expression revealed that the levels of the SASP components *Tnfa*, *Il1b* and *Il6* were significantly increased in aged mice (Fig. 2D). Furthermore, the expression of *iNOS* and *Mcp1* was also increased in the livers of aged mice (Fig. 2E). In contrast, the mRNA levels of *Arg1* and *Mrc1* decreased in aged mice, but there was no significant difference in the levels of *Il10* (Fig. 2F). Consistent with the results obtained from the gene transcription level, the content of the proinflammatory factors TNF-α, IL-1β, and IL-6 in the peripheral blood of aged mice increased, suggesting an anabatic SASP in aged mice during TAA-ALI (Fig. 2G). In addition, after administration of a higher dose of TAA, the survival curve showed that aged mice had higher mortality than the young mice (Fig. 2H, log-rank test *P* < 0.05). These results indicated that excessive and irreversible inflammation may be an essential factor of the poor prognosis of aged mice.

### Aging impairs autophagy in macrophages

**Fig. 3:**
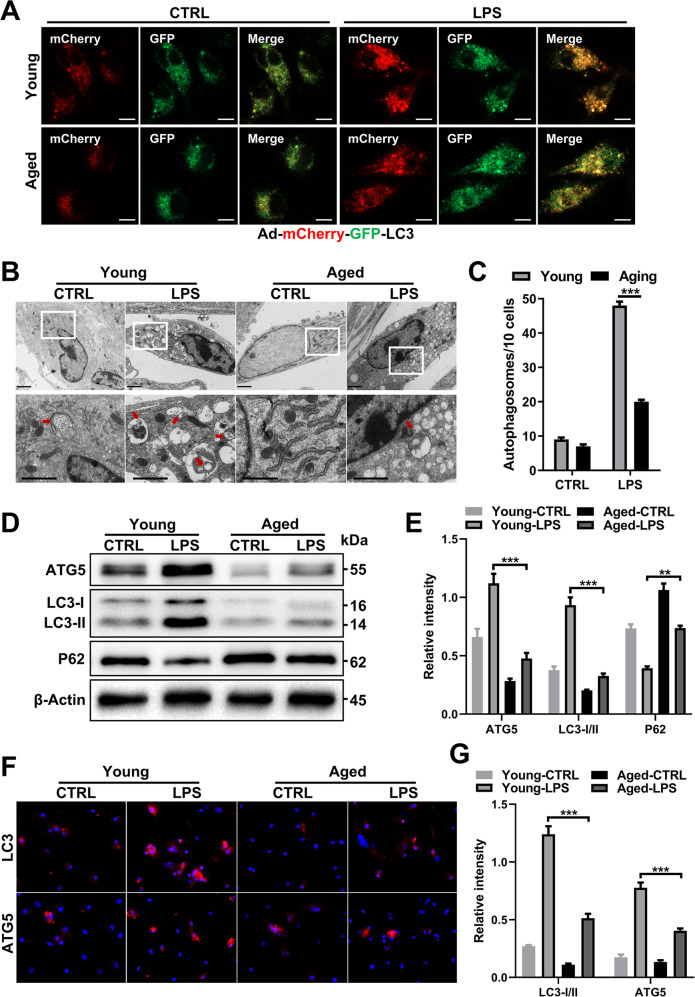
Aging impairs autophagy in LPS-treated macrophages. Young and aged BMDMs derived from mouse bone marrow were infected with Ad-mCherry-GFP-LC3 (20 MOI) on the 5th day when the cells were not fully mature and then treated with LPS (100 ng/ml) on the 7th day. **A** Representative laser confocal microscopy images. **B** Transmission electron microscopy (TEM) examination of autophagosomes from young and aged BMDMs and (**C**) quantification. **D** Immunoblotting of LC3-I/II, ATG5 and p62 expression in BMDMs from each group. Quantification in (**E**). **F** Representative IF staining of LC3-I/II and ATG5 incorporation (red fluorescence) with DAPI counterstaining (blue fluorescence) in BMDMs. Quantification in (**G**). All results are representative of at least three independent experiments. Values are presented as the mean ± SD. *p ≤ 0.05; **p ≤ 0.01; ***p ≤ 0.001.

Disparate polarization and inflammatory phenotypes were observed in macrophages during aging, and autophagy is an important component of the innate immune response. To understand the relationship of these phenotypes with autophagy, adenovirus expressing the mCherry-GFP-LC3 (Ad-mCherry-GFP-LC3) fusion protein was transfected into BMDMs from young and aged mice to evaluate autophagic flux by measuring turnover of the LC3 protein [22]. When mCherry is used to colabel LC3 with GFP, the acidic environment in lysosomes will lead to fluorescence quenching of GFP during the fusion of autophagosomes with lysosomes, while mCherry fluorescence is retained due to its stability. As shown in Fig. 3A, the autophagic flux of BMDMs from aged mice (aged BMDMs) was significantly lower than that of BMDMs from young mice (young BMDMs), which paralleled the impaired autophagy indicated by the reduced autophagosomes (Fig. 3B, C) and LC3 puncta (Supplementary Fig. S1A) in the cells after LPS treatment. Similarly, aging also reduced autophagy activation mediated by Torin 1 (Supplementary Fig. S1B).

Additionally, LPS induces the differentiation of macrophages to the M1 phenotype and can also induce autophagy [23]. Therefore, we further explored the influence of aging on the polarization phenotype of macrophages after LPS treatment, and autophagy-related proteins were detected by immunofluorescence (IF) and immunoblotting. The expression of ATG5 and LC3-II was decreased in aged BMDMs, and there was excessive intracellular accumulation of p62, an autophagy adapter protein, which can shuttle ubiquitinated cargo for autophagic degradation suggesting that autophagy was partially blocked in aged BMDMs (Fig. 3D, E). IF assays confirmed the above results, and the expression of LC3-II and ATG5 was decreased in aged cells (Fig. 3F, G, Supplementary S1A). In addition, Kupffer cells (KCs) were isolated from young and aged mice with or without TAA treatment. Consistent with the above in vitro results, aging decreased the autophagy level and ATG5 expression in KCs (Supplementary Fig. S1C).

### ATG5 restores autophagy and decreases the senescence phenotype

**Fig. 4:**
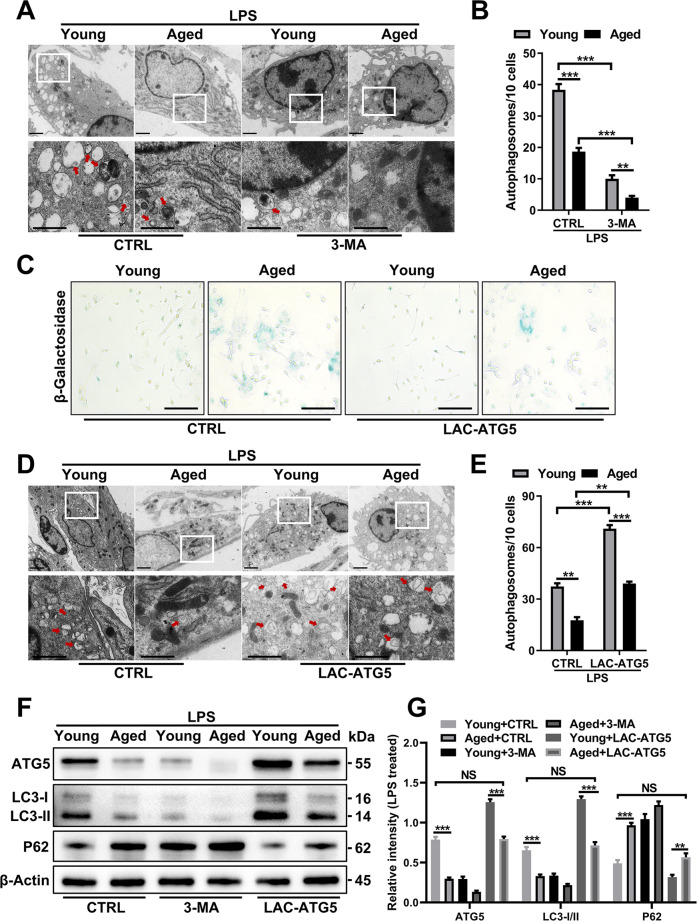
ATG5 restores autophagy and decreases the senescence phenotype. Young and aged BMDMs were treated with LPS (100 ng/ml) with or without pretreatment with 3-MA (5 mM) or LAC-ATG5 transfection. **A** With 3-MA pretreatment, TEM examination of autophagosomes from young and aged BMDMs. Quantification in (**B**). **C** Representative images of senescence-associated β-galactosidase staining. **D** TEM examination of autophagosomes from young and aged BMDMs after pretreatment with LAC-ATG5 and (**E**) quantification. **F** Immunoblotting of LC3-I/II, ATG5, and p62 expression in BMDMs from each group after pretreatment with 3-MA or LAC-ATG5 and (**G**) quantification. All results are representative of at least three independent experiments. Values are presented as the mean ± SD. *p ≤ 0.05; **p ≤ 0.01; ***p ≤ 0.001.

Next, BMDMs were pretreated with 3-MA before LPS treatment, and transmission electron microscopy (TEM) and IF images showed a further reduction in autophagosomes (Fig. 4A, B) and LC3 puncta (Supplementary Fig. S2) in aged BMDMs. To further study the role of ATG5 in aging, we transfected ATG5 lentiviral activation particles (LAC-ATG5) lentivirus into young and aged BMDMs. Senescence-associated β-galactosidase staining showed decreased β-galactosidase staining in the Aged-LAC-ATG5 group (Fig. 4C). TEM and IF examination showed an increased number of autophagosomes (Fig. 4D, E) and LC3 puncta (Supplementary Fig. S2) in aged BMDMs after ATG5 rescue. Immunoblotting confirmed these results, and LC3-II expression was upregulated in parallel (Fig. 4F, G). Notably, the autophagy level of aged macrophages after ATG5 overexpression was restored to a level comparable to that of normal young macrophages. In addition, P62 in aged BMDMs showed a remarkable decrease after ATG5 overexpression (Fig. 4F, G). In general, autophagy was impaired in aged macrophages due to the suppression of ATG5, and impaired autophagy may be a basic feature of aged macrophages.

### Aged macrophages preferentially polarize to the M1 phenotype

**Fig. 5:**
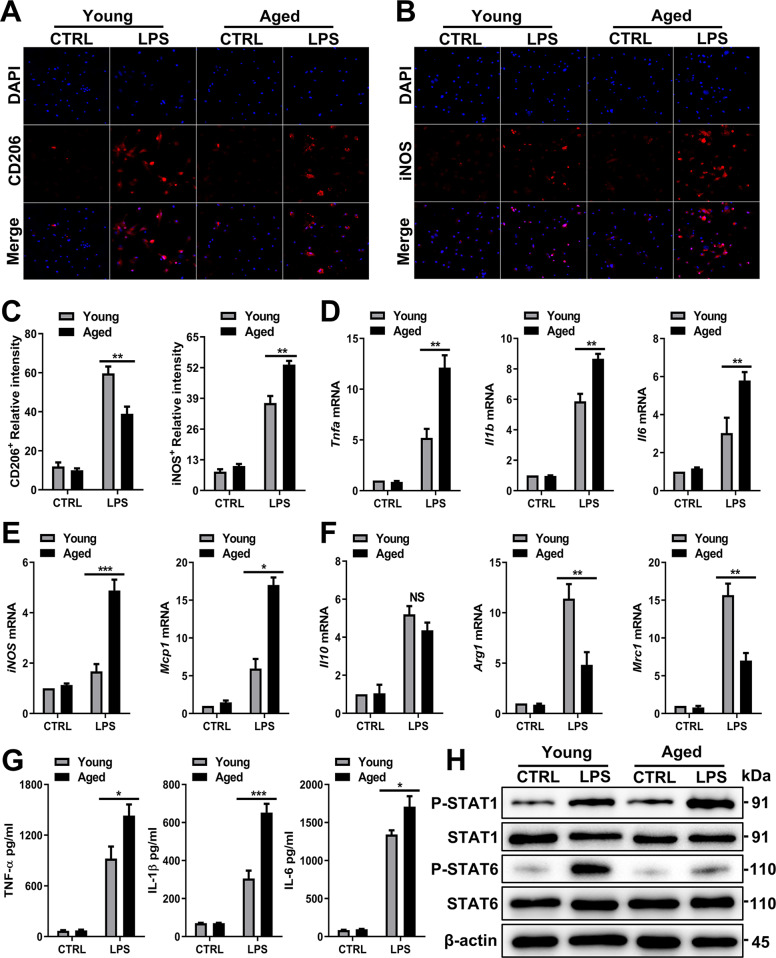
Aged macrophages preferentially polarize into the M1 phenotype. Young and aged BMDMs were treated with LPS (100 ng/ml) and PBS as a control. **A** Representative IF staining of CD206 incorporation (red fluorescence) in the liver with DAPI counterstaining (blue fluorescence). **B** Representative IF staining of iNOS incorporation (red fluorescence) in the liver with DAPI counterstaining (blue fluorescence). **C** Quantification of A (left) and B (right). **D**, **E** Proinflammatory mRNA expression (*Tnfa, Il1b, Il6, iNOS*, and *Mcp1*) and (**F**) anti-inflammatory mRNA expression (*Arg1, Il10*, and *Mrc1*) in BMDMs were detected by RT–PCR. The average target gene/GAPDH ratios of different experimental groups relative to the control group are given. **G** Determination of the total protein content (TNF-α, IL-1β, and IL-6) in the culture supernatant of BMDMs after LPS treatment by ELISA. **H** Immunoblotting of STAT1, P-STAT1, STAT6, and P-STAT6 expression in BMDMs from each group. All results are representative of at least three independent experiments. Values are presented as the mean ± SD. *p ≤ 0.05; **p ≤ 0.01; ***p ≤ 0.001.

Macrophages can be polarized to either a classical (M1) or an alternative (M2) activation state [24]. Indeed, compared to young BMDMs, aged BMDMs exhibited decreased levels of CD206 (an M2 marker) (Fig. 5A, C) but increased levels of iNOS (an M1 marker) (Fig. 5B, C). Higher-resolution images of each cell are shown in Supplemetary Fig. S3C. Immunoblotting examination of KCs (Supplementary Fig. S1D) and BMDMs confirmed these results (Supplementary Fig. S3A). The mRNA expression of *Tnfa, Il1b, Il6, iNOS, Mcp1, Arg1, Il10*, and *Mrc1* was measured next. The expression of the proinflammatory cytokines and mediators *Tnfa, Il1b, Il6, iNOS*, and *Mcp1* (M1 phenotype) was increased in aged BMDMs (Fig. 5D, E), but the expression of the anti-inflammatory mediators *Arg1* and *Mrc1* (M2 phenotype) was decreased (Fig. 5F). Consistent with the mRNA expression results, higher levels of TNF-α, IL-1β, and IL-6 were secreted by aged BMDMs (Fig. 5G).

Moreover, STAT1 is highly activated in proinflammatory macrophages (induced by LPS/IFN-γ, which induce an acute inflammatory response) [25], while STAT6 is highly activated in anti-inflammatory macrophages (induced by IL-4, which resolves inflammation) [26, 27]. We found that aged BMDMs visibly activated STAT1 phosphorylation and inhibited STAT6 phosphorylation (Fig. 5H). Collectively, these findings show that aged macrophages preferentially polarize to the proinflammatory M1 phenotype.

### Impaired autophagy promotes increased M1 polarization of aged macrophages compared to young macrophages

**Fig. 6:**
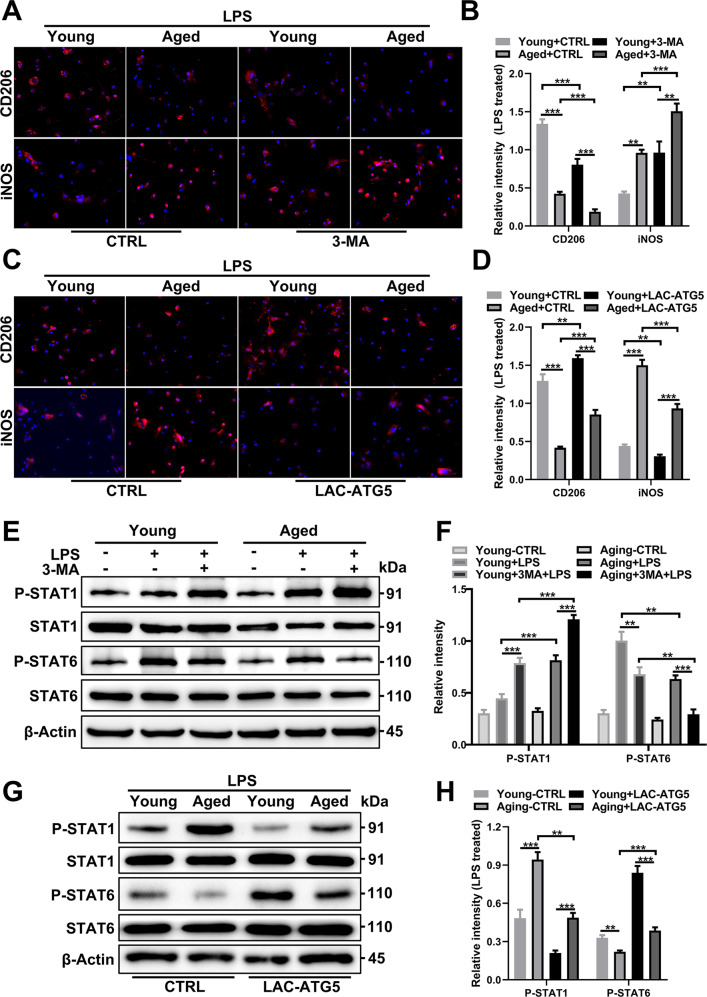
Autophagy alters macrophage polarization in aged and young mice. Young and aged BMDMs were treated with LPS (100 ng/ml) with or without pretreatment with 3-MA (5 mM) or LAC-ATG5. **A** Representative IF staining of CD206 and iNOS incorporation (red fluorescence) with DAPI counterstaining (blue fluorescence) in BMDMs from young and aged BMDMs after 3-MA pretreatment. Quantification in (**B**). **C** Representative IF staining of CD206 and iNOS incorporation (red fluorescence) with DAPI counterstaining (blue fluorescence) in BMDMs from each group after pretreatment with LAC-ATG5. Quantification in (**D**). **E** Immunoblotting of STAT1, P-STAT1, STAT6, and P-STAT6 expression in young and aged BMDMs was performed after 3-MA pretreatment. Quantification in (**F**). **G** Immunoblotting of STAT1, P-STAT1, STAT6, and P-STAT6 expression in BMDMs from each group was performed after pretreatment with LAC-ATG5. Quantification in (**H**). All results are representative of at least three independent experiments. Values are presented as the mean ± SD. *p ≤ 0.05; **p ≤ 0.01; ***p ≤ 0.001.

Compared with treatment with LPS alone, treatment of BMDMs from both young and aged mice with LPS combined with 3-MA induced a higher proportion of iNOS^+^ cells (M1) and a lower proportion of CD206^+^ cells (M2), indicating that macrophages were polarized to the M1 phenotype after inhibition of autophagy, and this trend was more pronounced in aged BMDMs (Fig. 6A, B). Next, we explored how increased ATG5 expression affects macrophage polarization. The proportion of iNOS^+^ cells was decreased in aged BMDMs after ATG5 overexpression and almost identical to that of young mice without ATG5 transfection (Fig. 6C, D). Analogously, higher-resolution images of each cell are shown in Supplementary Fig. S3C, and immunoblotting examination of BMDMs (Supplementary Fig. S3B) from each experimental group confirmed these results. Further, we found that Torin 1 also resulted in an increase in autophagy level and a decrease in M1 polarization similar to that achieved by ATG5 rescue (Supplementary Fig. S3D, E).

In addition, STAT1 showed increased phosphorylation in aged BMDMs treated with LPS, but STAT6 showed decreased phosphorylation. 3-MA addition further promoted STAT1 activation and STAT6 suppression (Fig. 6E, F), while ATG5 expression reduced STAT1 activation and promoted STAT6 activation (Fig. 6G, H). Together, these findings show that aging-dependent autophagy deficiency promotes M1 polarization via ATG5 repression, and ATG5 enhancement can restore the polarization phenotype to that observed under youthful conditions.

### M1 macrophages exacerbate liver inflammation and TAA-ALI, while M2 macrophages almost have the opposite effect

**Fig. 7:**
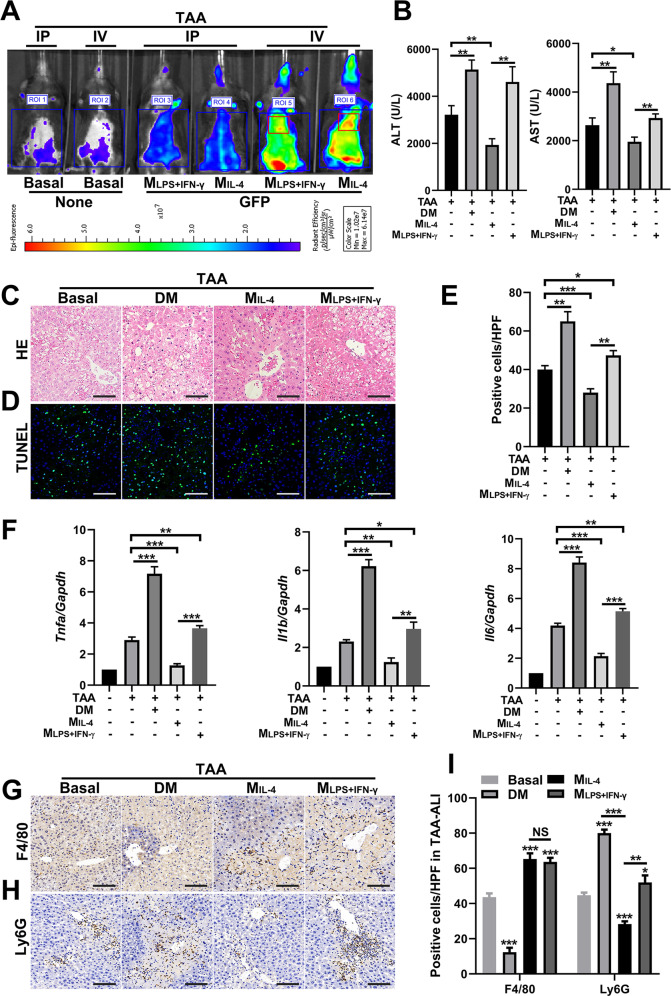
IL-4-induced BMDMs show reduced necrosis and apoptosis following TAA-ALI. IL-4 or LPS + IFN-γ was used to treat GFP-labeled BMDMs, and the cells were transplanted by intraperitoneal injection (IP) or tail vein injection (IV). **A** Representative images of macrophage movement after transplantation using an in vivo imaging system. Next, we performed the following experimental treatments before TAA-ALI (200 mg/kg) was established: macrophage depletion by clodronate liposome injection (DM), transplantation of LPS + IFN-γ-induced BMDMs (M_LPS+IFN-γ_), and transplantation of IL-4-induced BMDMs (M_IL-4_). The results of the TAA-ALI groups are shown here, and the control (PBS-treated) groups are shown in S4. **B** Serum ALT (left panel) and AST (right panel) levels in each group. **C** Representative H&E staining of the liver. **D** Representative TUNEL (green fluorescence) staining of the liver with DAPI counterstaining (blue fluorescence) and (**E**) quantification. **F** mRNA expression (*Tnfa, Il1b, Il6*) in liver tissue was detected by RT–PCR. The average target gene/GAPDH ratios of different experimental groups relative to the control group are given. (**G**, **H**) Representative F4/80 and (H) Ly6G IHC staining images of liver tissue from each group. **I** Quantification of G and H. All data shown represent *n* = 8–10 mice per group. All results are representative of at least three independent experiments. Values are presented as the mean ± SD. *p ≤ 0.05; **p ≤ 0.01; ***p ≤ 0.001.

A fully defined protocol to generate M1 (LPS- and IFN-γ-induced macrophages, M_LPS+IFN-γ_) and M2 (M_IL-4_-induced macrophages, M_IL-4_) macrophages has been described [28, 29]. Here, we artificially polarized BMDMs into M_LPS+IFN-γ_ and M_IL-4_ phenotypes for transplantation, and 8-week-old mice were chosen as BMDM donors and recipients. Specifically, we transplanted GFP-labeled M_LPS+IFN-γ_ and M_IL-4_ cells into mice via intraperitoneal injection (IP) and caudal vein injection (IV), and the TAA-ALI model was established 12 h after cell transplantation. Using an in vivo imaging system, we detected the migratory sites of GFP-labeled M_LPS+IFN-γ_ and M_IL-4_ cells. We found that macrophages transplanted via the caudal vein aggregated to the liver during TAA-ALI, whereas intraperitoneal injection did not result in significant fluorescence enhancement in the liver region (Fig. 7A). Moreover, both M_IL-4_ and M_LPS+IFN-γ_ cells aggregated in the liver after TAA treatment, and the fluorescence intensity of M_IL-4_ aggregation was higher (Fig. 7A).

We next established the following four pretreatments for mice, with further administration of TAA 12 h later: depletion of macrophages with clodronate liposomes (DM), transplantation of M_LPS+IFN-γ_ macrophages, transplantation of M_IL-4_ macrophages, and the control. Then, liver injury and inflammation were measured in each group. ALT and AST levels were significantly increased in the DM group, which paralleled the liver injury seen by H&E, indicating that macrophages play an essential role in antagonizing liver injury (Fig. 7B, C; Supplementary S4A, B). TUNEL staining confirmed these results, and mice from the DM group exhibited a significantly increased area of hepatic necrosis compared to that of the control group (Fig. 7D, E; Supplementary S4C). Meanwhile, transplantation of M_IL-4_ cells significantly alleviated liver injury, reducing serum ALT and AST levels and hepatocyte necrosis, which were accentuated by M_LPS+IFN-γ_ transplantation (Fig. 7B, C). In addition, hepatocyte apoptosis was aggravated in the DM and M_LPS+IFN-γ_ groups, but it was attenuated in the M_IL-4_ group, indicating that M1 macrophages have an exacerbating effect on ALI, while M2 macrophages have a somewhat protective effect (Fig. 7D, E).

Given the association between inflammation and liver injury, we further examined the expression of cytokines and the infiltration of macrophages and neutrophils. The expression of *Tnfa, Il1b*, and *Il6* increased significantly in the DM group during TAA-ALI, indicating that macrophage deficiency causes dysregulation of inflammatory factors, thus aggravating liver injury (Fig. 7F; Supplementary S4D). Macrophage infiltration was increased in both the M_LPS+IFN-γ_ and M_IL-4_ transplantation groups, and but sparsely in the DM group (Fig. 7G, I; Supplementary S4E). Consistent with the severe liver injury observed in the DM group, abundant neutrophils infiltrated the liver. Notably, M_IL-4_ transplantation significantly reduced neutrophil recruitment to the liver, while more neutrophils were present in the M_LPS+IFN-γ_ group than in control, suggesting that M1 macrophages promote neutrophil infiltration, thereby aggravating inflammation and liver injury, but M2 macrophages inhibit neutrophil infiltration, thereby accelerating inflammation resolution and tissue repair (Fig. 7H, I; Supplementary S4F).

### ATG5 rescue in aged macrophages contributes to the remission of TAA-ALI

**Fig. 8:**
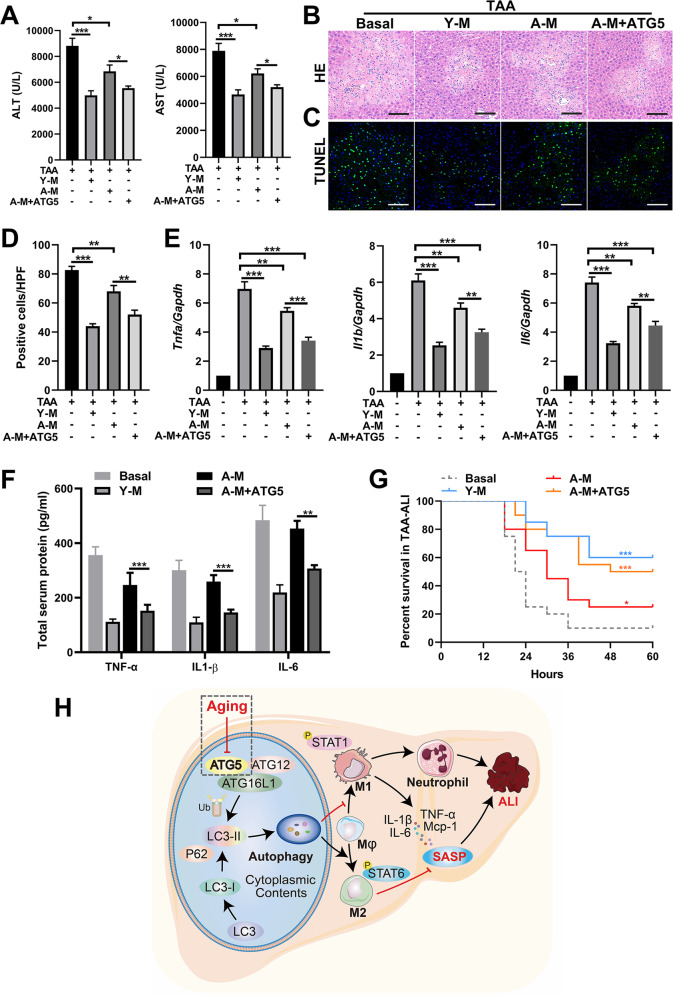
Administration of BMDMs with ATG5 overexpression relieves TAA-ALI. Young BMDMs (Y-M) and aged BMDMs (A-M) were isolated and from young and aged mice. Three macrophage populations derived from the mouse BM and the control were prepared: Y-M, A-M, and A-M with LAC-ATG5 (A-M + ATG5). After transplantation of these three kinds of BMDMs by tail vein injection, a TAA-ALI model (200 mg/kg) was established. The results of the transplantation of A-M treated with Torin 1 are shown in S5. **A** Serum ALT (left panel) and AST (right panel) levels in each group. **B** Representative H&E staining of the liver. **C** Representative TUNEL (green fluorescence) staining of the liver with DAPI counterstaining (blue fluorescence) and (**D**) quantification. **E** mRNA expression (*Tnfa, Il1b*, and *Il6*) in the liver tissue was detected by RT–PCR. The average target gene/GAPDH ratios of different experimental groups relative to the control group are given. **F** Determination of the total protein content (TNF-α, IL-1β, and IL-6) in the serum by ELISA. Then, a TAA dose of 500 mg/kg was used to calculate mortality. **G** Summary of mouse mortality in each group. **H** Model depicting the aggravation of ALI during aging via altered macrophage polarization induced by autophagy deficiency. During aging, macrophage autophagy decreases in mice, which may be ascribed to the downregulation of ATG5. In addition, macrophages are preferentially polarized to the M1 phenotype in ALI, accompanied by increased production of proinflammatory factors (SASP) and massive neutrophil infiltration, thus aggravating ALI. In addition, this aggravation can be reversed by transplantation of macrophages with ATG5 restoration. All data shown represent *n* = 8–10 mice per group, and 20 mice per group were used for mortality analysis. All results are representative of at least three independent experiments. Values are presented as the mean ± SD. *p ≤ 0.05; **p ≤ 0.01; ***p ≤ 0.001.

To investigate whether ATG5 rescue in macrophages can restore resistance to liver injury in aged mice, young BMDMs (Young-M), aged BMDMs (A-M) and ATG5-overexpressing A-M (A-M + ATG5) were transplanted into aged mice, and TAA-ALI was performed 12 h later. The serum ALT and AST levels decreased in all macrophage transplantation groups, and the A-M + ATG5 group had lower levels than the A-M group, while the lowest levels were observed in the Y-M group (Fig. 8A; Supplementary S5A), which was paralleled by the liver injury seen with H&E (Fig. 8B; Supplementary S5B). Consistent with these findings, TUNEL staining showed that ATG5 rescue reduced the quantity of apoptotic hepatocytes compared to that in the A-M group (Fig. 8C, D; Supplementary S5C). In addition, the expression of proinflammatory cytokines (*Tnfa, Il1b*, and *Il6*) in all BMDM transplantation groups decreased (Fig. 8E). Notably, the greatest decrease was observed in the Y-M group, the smallest decrease was observed in the A-M group, and an intermediate decrease was observed in the A-M + ATG5 group (Fig. 8E; Supplementary S5D), which was paralleled by the serum total protein levels of TNF-α, IL-1β and IL-6 (Fig. 8F).

Interestingly, activation of autophagy in aged macrophages by Torin1 also has a similar positive effect on TAA-ALI, with overexpression of ATG5, reduction of AST and ALT levels (Supplementary Fig. S5A), alleviation of hepatocyte apoptosis and necrosis (Supplementary Fig. S5B, C), and downregulation of liver proinflammatory factors (Supplementary Fig. S5D).

We then increased the dose of TAA (500 mg/kg) to investigate the effect of ATG5 rescue on mortality in TAA-ALI. Mice without macrophage transplantation had the highest mortality, and the mice in the Y-M group had the lowest mortality (log-rank test *P* < 0.001), while the mortality of the mice in the A-M + ATG5 group was reduced compared to that of the mice in the A-M group (log-rank test *P* < 0.05) and was almost identical to that of the mice in the Y-M group (Fig. 8G). In brief, ATG5 could restore autophagy in aged macrophages and moderate the proinflammatory phenotype.

## Discussion

Autophagy is a protein-decluttering process that is highly conserved and present in all eukaryotes known to date [30]. Recent studies have suggested a new immune regulation mechanism in which autophagy modulates innate immunity by influencing the polarization of M1 and M2 macrophages [31]. By regulating the fate of hematopoietic stem cells, the recruitment of monocytes, and the differentiation of macrophages, autophagy plays a role in the production and development of macrophages [32]. Enhancing autophagy in macrophages can reduce macrophage apoptosis and IL-1β levels, thereby reducing atherosclerosis [33]. The present study examined the effect of aging on autophagy in macrophages during TAA-ALI, confirming that autophagy is impaired in macrophages and TAA-ALI is aggravated in aged mice. In addition, we found that impaired autophagy in aged macrophages was triggered by ATG5 repression.

Aging is characterized by a persistent proinflammatory response and a stable cell cycle, and it is an irreversible process leading to atherosclerosis, metabolic syndrome, cancer, and poor disease prognosis [34, 35]. The aged liver is also susceptible to inflammation, as evidenced by the high incidence of aging-related cirrhosis, and cancer [36]. Increasing evidence shows that many mechanisms that regulate lifespan extension, such as calorie restriction, require the participation of autophagy in various organisms [37]. The current study demonstrates that ATG5 deficiency leads to decreased autophagy and regulates the polarization of macrophages during aging.

The liver has a strong capacity to regenerate, but aging greatly weakens this ability, leading to the deteriorative events of acute injury, cirrhosis and cancer [38, 39]. Although the pathogenesis of liver disorders, including ALI, ALF, and fibrosis, remains poorly understood, inflammation appears to play an indispensable role. The activation of macrophages produces proinflammatory and vascular permeability mediators, induces the accumulation of neutrophils, lymphocytes, eosinophils, and monocytes in the liver, and promotes tissue injury [40]. Our findings mirror those seen following injury using animal models for hepatic fibrosis, where macrophages have been shown to be responsible for driving inflammation and disrupting the regenerative capacity of senescent organs [41]. The release of SASP factors, such as IL-1β, IL-6, TNF-α, and MCP-1, activates the innate immune system, recruits neutrophils to the liver, and finally leads to hepatocyte death due to the dysregulated inflammatory response. Thus, SASP control is particularly necessary for liver regeneration and functional recovery during aging.

In conclusion, senescence is accompanied by the senescence of macrophages; this senescence is manifested by decreased autophagy, increased M1 polarization, and increased secretion of the proinflammatory SASP. Autophagy recovery via ATG5 rescue in aged macrophages modulated macrophage polarization and the cytokine secretion phenotype (Fig. 8H). Moreover, further in-depth study of the relationship between autophagy and aging, as well as the underlying regulatory mechanism, may improve our understanding of the lifespan and promote longevity.

## Materials and methods

### Animals and models

Young (6–10 weeks) and aged (> 16 months) [42] male C57BL/6 N mice were used. All mouse experiments were approved by the Laboratory Animal Ethics Committee of Anhui Medical University. Mice were placed in an environmentally controlled, pathogen-free isolation facility under a 12 h light-dark cycle, and food and water were freely available. In the functional experiment, the mice were randomly divided into experimental and control groups, and TAA was administered to the experimental mice at a dose of 200 mg/kg for 24 h. In the mortality experiment, TAA was administered to the experimental mice at a higher dose of 500 mg/kg, and the time and number of deaths were observed and recorded.

### Culture and differentiation of primary BMDMs

Bone marrow cells were isolated from the femurs and tibias of young and aged mice. After RBC removal, the cells were cultured in DMEM containing 10% FBS and 20 ng/ml M-CSF (PeproTech) for 7 days.

Differentiation induction of M1 and M2 macrophages: Cells were seeded and allowed to adhere overnight at 37 °C in 5% CO_2_. The following day, the cells were treated with an M1 (M_LPS+IFN-γ_) cocktail that consisted of LPS (100 ng/mL) and IFN-γ (20 ng/mL) and an M2 (M_IL-4_) cocktail that consisted of IL-4 (20 ng/mL) [43]. All cocktails were diluted in fresh medium. The cells were incubated for 18 h to obtain sufficient polarization.

### Isolation of mouse KCs

Mouse livers were perfused in situ via the portal vein with Hanks balanced salt solution (HBSS, Gibco), followed by 0.3% collagenase IV (Sigma-Aldrich). The perfused tissues were minced into pieces, and the fluid was discharged onto a 70 μm filter (BD Biosciences) that had been prewetted with 1 ml of PBS containing 0.5% bovine serum albumin (BSA, Sigma-Aldrich) over a 50-ml conical tube. The cell suspension was centrifuged at a speed of 500 g for 4 min at 4 °C. The pellet was resuspended in 20 mL of 35% Percoll and then layered on a 67% Percoll gradient (13 ml), followed by centrifugation at 600 g for 20 min at 4 °C. Nonparenchymal cells (KCs) from the interface between the two density cushions of 35% with 67% Percoll were then resuspended in PBS followed by centrifugation at 1600 RPM for 10 min. Then, the cells were cultured in DMEM supplemented with 10% FBS at 37 °C for 1 h, and the nonadherent cells were removed. The adherent cells were used for further experiments.

### Biochemical assays

Mouse peripheral blood was collected and centrifuged to obtain serum for analysis. We used a Mairui automated biochemical analyzer (BS-350E) to detect serum alanine aminotransferase (ALT) and aspartate aminotransferase (AST).

### Hematoxylin-Eosin staining

Fresh liver tissue was soaked in formalin, dehydrated with alcohol and cleared with xylene. Then, the paraffin-embedded liver tissue was cut into thin slices of a suitable thickness, placed on glass slides, dewaxed in water, stained with hematoxylin-eosin, and sealed with resin gel.

### Histopathology

Liver sections of a 4 µm thickness that were fixed in 4% paraformaldehyde buffered with PBS were used for staining. After deparaffinization, the sections were washed in PBS three times. EDTA buffer and Dako blocking reagent were used for heat-induced antigen retrieval and blocking of endogenous peroxidase activity. Primary antibody incubation was performed at 4 °C overnight with shaking, followed by streptavidin-conjugated horseradish peroxidase antibody incubation at room temperature for 2 h. DAB staining was performed, and the sections were washed with distilled water, followed by hematoxylin counterstaining.

### Immunofluorescence

Cells were fixed on slides with 4% paraformaldehyde for 10 min. After washing with PBS three times, the cells were permeabilized with 0.5% Triton X-100 in PBS for 10 min. After rinsing the cells with PBS, with cells were blocked with PBS containing 0.025% Triton X-100 and 1% BSA for 1 h at room temperature. The slides were then incubated with the primary antibodies in 0.025% Triton X-100 and 1% BSA in PBS at 4 °C overnight. Then, the slides were washed with PBS with 0.025% Triton X-100 three times and incubated with fluorochrome-conjugated secondary antibody and DAPI for 1 h. The images were obtained on a Zeiss Axio Observer 3 imaging system or Zeiss LSM 800 confocal laser scanning microscope.

### Immunoblotting

Total protein extraction was performed with RIPA buffer supplemented with a complete protease inhibitor cocktail. The lysates were clarified by centrifugation at 12,000 rpm for 15 min at 4 °C, and the concentration was measured by a Nanodrop 2000 (Thermo Fisher). The lysate was denatured at 100 °C for 10 min and then mixed with a loading buffer to 1X. Protein extracts (5–10 μl) were separated on 10% SDS-polyacrylamide gels and transferred onto PVDF membranes. Then, blocking with 5% nonfat milk in TBST (20 mM Tris base, 137 mM NaCl, 0.1% Tween 20, pH 7.4) was performed for 1 h at room temperature. The membrane was incubated overnight at 4 °C in a 5% BSA-TBST solution containing the following primary antibodies: LC3-I/II (CST, 12741), ATG5 (CST, 12994), P62 (CST, 16177), Bax (CST, 14796), Bcl-2 (CST, 3498), iNOS (CST, D6B6S), CD206 (Santa Cruz, sc-70586), P-STAT1 (CST, 8062), P-STAT6 (CST, 56554), STAT1 (CST, 9172), STAT6 (CST, 9362), and β-actin (CST, 4970).

### RNA isolation, RT–PCR analysis, and lentiviral activation particles

Total RNA was extracted using TRIzol (Invitrogen). We synthesized cDNA from 1 μg of total RNA using the manufacturer’s protocol (Takara, RR036A). Then, RT–PCR was conducted using a premixed kit (Takara, RR820A) according to the vendor’s manual. The sequences of the primer pairs are listed in Supplementary Table S1. ATG5 lentiviral activation particles (sc-419149-LAC) and control lentiviral activation particles (sc-437282) were purchased from Santa Cruz Biotechnology, Inc. Lentiviral activation particle transduction was performed following the instructions provided by the manufacturer.

### Ad-mCherry-GFP-LC3

Ad-mCherry-GFP-LC3 (Beyotime Biotechnology, C3011), which is adenovirus expressing the mCherry-GFP-LC3 fusion protein, can be used for autophagy detection after infection of cells or tissues. BMDMs were infected with Ad-mCherry-GFP-LC3 (20 MOI) according to the manufacturer’s instructions on the 5th day when the cells were not fully mature, and then treated with LPS or Torin 1 (1 µM) on the 7th day.

### Statistical analysis

Statistical analysis was performed with Prism 8.2 (GraphPad Software). To test two groups, an unpaired two-tailed t-test or the Mann-Whitney U-test was performed on parametric or nonparametric datasets, respectively. To test more than two groups, one-way ANOVA, two-way ANOVA (with Dunnett’s multiple comparison test), or a mixed-effects model (with Sidak’s multiple comparisons test) was performed. The differences in the survival curves were analyzed by the log-rank test. Differences for which *P* < 0.05 were considered statistically significant.

## Supplementary information

Supplemental Information

## Data Availability

The data generated or analyzed during this study are included in this published article and its supplementary information files.

## References

[1] Forbes SJ, Newsome PN (2016). Liver regeneration - mechanisms and models to clinical application. Nat Rev Gastroenterol. Hepatol.

[2] Michalopoulos GK (2017). Hepatostat: liver regeneration and normal liver tissue maintenance. Hepatology.

[3] Tujios SR, Lee WM (2018). Acute liver failure induced by idiosyncratic reaction to drugs: challenges in diagnosis and therapy. Liver Int.

[4] Kan C, Ungelenk L, Lupp A, Dirsch O, Dahmen U (2018). IschemIa-reperfusion Injury In Aged Livers-the Energy Metabolism, Inflammatory Response, And Autophagy. Transplantation.

[5] Brenner C, Galluzzi L, Kepp O, Kroemer G (2013). Decoding cell death signals in liver inflammation. J Hepatol.

[6] Krenkel O, Tacke F (2017). Liver macrophages in tissue homeostasis and disease. Nat Rev Immunol.

[7] Kubes P, Jenne C (2018). Immune responses in the liver. Annu Rev Immunol.

[8] Nikolich-Zugich J (2018). The twilight of immunity: emerging concepts in aging of the immune system. Nat Immunol.

[9] Lopez BG, Tsai MS, Baratta JL, Longmuir KJ, Robertson RT (2011). Characterization of Kupffer cells in livers of developing mice. Comp Hepatol.

[10] Jackaman C, Tomay F, Duong L, Abdol Razak NB, Pixley FJ, Metharom P (2017). Aging and cancer: the role of macrophages and neutrophils. Ageing Res Rev.

[11] Locati M, Curtale G, Mantovani A (2020). Diversity, mechanisms, and significance of macrophage plasticity. Annu Rev Pathol.

[12] Reidy PT, McKenzie AI, Mahmassani ZS, Petrocelli JJ, Nelson DB, Lindsay CC (2019). Aging impairs mouse skeletal muscle macrophage polarization and muscle-specific abundance during recovery from disuse. Am J Physiol Endocrinol Metab.

[13] Stahl EC, Haschak MJ, Popovic B, Brown BN (2018). Macrophages in the aging liver and age-related liver disease. Front Immunol.

[14] Ni HM, McGill MR, Chao X, Du K, Williams JA, Xie Y (2016). Removal of acetaminophen protein adducts by autophagy protects against acetaminophen-induced liver injury in mice. J Hepatol.

[15] Ilyas G, Zhao E, Liu K, Lin Y, Tesfa L, Tanaka KE (2016). Macrophage autophagy limits acute toxic liver injury in mice through down regulation of interleukin-1beta. J Hepatol.

[16] Han J, Bae J, Choi CY, Choi SP, Kang HS, Jo EK (2016). Autophagy induced by AXL receptor tyrosine kinase alleviates acute liver injury via inhibition of NLRP3 inflammasome activation in mice. Autophagy.

[17] Kim SH, Kim G, Han DH, Lee M, Kim I, Kim B (2017). Ezetimibe ameliorates steatohepatitis via AMP activated protein kinase-TFEB-mediated activation of autophagy and NLRP3 inflammasome inhibition. Autophagy.

[18] Lodder J, Denaes T, Chobert MN, Wan J, El-Benna J, Pawlotsky JM (2015). Macrophage autophagy protects against liver fibrosis in mice. Autophagy.

[19] Shirato T, Homma T, Lee J, Kurahashi T, Fujii J (2017). Oxidative stress caused by a SOD1 deficiency ameliorates thioacetamide-triggered cell death via CYP2E1 inhibition but stimulates liver steatosis. Arch Toxicol.

[20] Deng X, Zhang X, Li W, Feng RX, Li L, Yi GR (2018). Chronic liver injury induces conversion of biliary epithelial cells into hepatocytes. Cell Stem Cell.

[21] Bai Y, Bai Y, Wang S, Wu F, Wang DH, Chen J (2018). Targeted upregulation of uncoupling protein 2 within the basal ganglia output structure ameliorates dyskinesia after severe liver failure. Free Radic Biol Med.

[22] Kaizuka T, Morishita H, Hama Y, Tsukamoto S, Matsui T, Toyota Y (2016). An autophagic flux probe that releases an internal control. Mol Cell.

[23] Xu Y, Jagannath C, Liu XD, Sharafkhaneh A, Kolodziejska KE, Eissa NT (2007). Toll-like receptor 4 is a sensor for autophagy associated with innate immunity. Immunity.

[24] Becker L, Nguyen L, Gill J, Kulkarni S, Pasricha PJ, Habtezion A (2018). Age-dependent shift in macrophage polarisation causes inflammation-mediated degeneration of enteric nervous system. Gut.

[25] Ma J, Wei K, Liu J, Tang K, Zhang H, Zhu L (2020). Glycogen metabolism regulates macrophage-mediated acute inflammatory responses. Nat Commun.

[26] Iwata H, Goettsch C, Sharma A, Ricchiuto P, Goh WW, Halu A (2016). PARP9 and PARP14 cross-regulate macrophage activation via STAT1 ADP-ribosylation. Nat Commun.

[27] Begitt A, Cavey J, Droescher M, Vinkemeier U (2018). On the role of STAT1 and STAT6 ADP-ribosylation in the regulation of macrophage activation. Nat Commun.

[28] Chen S, Kammerl IE, Vosyka O, Baumann T, Yu Y, Wu Y (2016). Immunoproteasome dysfunction augments alternative polarization of alveolar macrophages. Cell Death Differ.

[29] Herd HL, Bartlett KT, Gustafson JA, McGill LD, Ghandehari H (2015). Macrophage silica nanoparticle response is phenotypically dependent. Biomaterials.

[30] Escobar KA, Cole NH, Mermier CM, VanDusseldorp TA (2019). Autophagy and aging: maintaining the proteome through exercise and caloric restriction. Aging Cell.

[31] Liu K, Zhao E, Ilyas G, Lalazar G, Lin Y, Haseeb M (2015). Impaired macrophage autophagy increases the immune response in obese mice by promoting proinflammatory macrophage polarization. Autophagy.

[32] Chen P, Cescon M, Bonaldo P (2014). Autophagy-mediated regulation of macrophages and its applications for cancer. Autophagy.

[33] Sergin I, Evans TD, Zhang X, Bhattacharya S, Stokes CJ, Song E (2017). Exploiting macrophage autophagy-lysosomal biogenesis as a therapy for atherosclerosis. Nat Commun.

[34] Franceschi C, Capri M, Monti D, Giunta S, Olivieri F, Sevini F (2007). Inflammaging and anti-inflammaging: a systemic perspective on aging and longevity emerged from studies in humans. Mech Ageing Dev.

[35] Ferrucci L, Fabbri E (2018). Inflammageing: chronic inflammation in ageing, cardiovascular disease, and frailty. Nat Rev Cardiol.

[36] Amor C, Feucht J, Leibold J, Ho YJ, Zhu C, Alonso-Curbelo D (2020). Senolytic CAR T cells reverse senescence-associated pathologies. Nature.

[37] Shirakabe A, Ikeda Y, Sciarretta S, Zablocki DK, Sadoshima J (2016). Aging and autophagy in the heart. Circ Res.

[38] Chung TW, Kim EY, Han CW, Park SY, Jeong MS, Yoon D, et al. Machilin A inhibits tumor growth and macrophage M2 polarization through the reduction of lactic acid. Cancers (Basel). 2019;11(7):963. 10.3390/cancers11070963 PMC6678097 31324019

[39] Maeso-Diaz R, Ortega-Ribera M, Lafoz E, Lozano JJ, Baiges A, Frances R (2019). Aging influences hepatic microvascular biology and liver fibrosis in advanced chronic liver disease. Aging Dis.

[40] Bernsmeier C, van der Merwe S, Perianin A (2020). Innate immune cells in cirrhosis. J Hepatol.

[41] Chen Y, Pu Q, Ma Y, Zhang H, Ye T, Zhao C (2021). Aging reprograms the hematopoietic-vascular niche to impede regeneration and promote fibrosis. Cell Metab.

[42] Ramirez T, Li YM, Yin S, Xu MJ, Feng D, Zhou Z (2017). Aging aggravates alcoholic liver injury and fibrosis in mice by downregulating sirtuin 1 expression. J Hepatol.

[43] Huleihel L, Dziki JL, Bartolacci JG, Rausch T, Scarritt ME, Cramer MC (2017). Macrophage phenotype in response to ECM bioscaffolds. Semin Immunol.

