# Supplementary material for: Autophagy deficiency promotes M1 macrophage polarization to exacerbate acute liver injury via ATG5 repression during aging
Source: Cell Death Discov. 2021 Dec 20;7:397. doi: 10.1038/s41420-021-00797-2 (PMC8688512; doi:10.1038/s41420-021-00797-2)
Supplement: Supplementary file 1 — Supplemental Information [file 41420_2021_797_MOESM1_ESM.docx]

**Supplemental Information**

**Title: Autophagy deficiency promotes M1 macrophage polarization to exacerbate acute liver injury via ATG5 repression during aging**

Rui Liu^1,2^, Juanjuan Cui^3^, Yating Sun^4^, Wentao Xu^1,5^, Ziming Wang^1^, Miaomiao Wu^2^, Huke Dong^5^, Congcong Yang^4^, Shaocheng Hong^5^, Shi Yin^6^, Hua Wang^1,2*^

^1^Department of Oncology, the First Affiliated Hospital of Anhui Medical University, Hefei 230022, China

^2^Inflammation and Immune Mediated Diseases Laboratory of Anhui Province, Anhui Medical University, Hefei 230032, China

^3^Department of Stomatology, the First Affiliated Hospital of Anhui Medical University, Hefei 230022, China

^4^Department of Genetics, School of Life Science, Anhui Medical University, Hefei, Anhui, 230032, China

^5^First Clinical Medical College of Anhui Medical University, Hefei 230036

^6^Department of Geriatrics, the First Affiliated Hospital of USTC, Division of Life Sciences and Medicine, University of Science and Technology of China, Hefei, Anhui 230001, China

*Correspondence: Hua Wang, M.D., Professor

Department of Oncology, the First Affiliated Hospital of Anhui Medical University, 218 Jixi Road, Hefei, Anhui Province, 230022, PR China. Tel/fax: +86-551-65161056. E-mail address: wanghua@ahmu.edu.cn (H. Wang).

Rui Liu, Juanjuan Cui and Yating Sun contributed equally in this study.

Conflict of interest: The Authors declare that they have no conflict of interest.

**Supplementary Figure 1.**

**
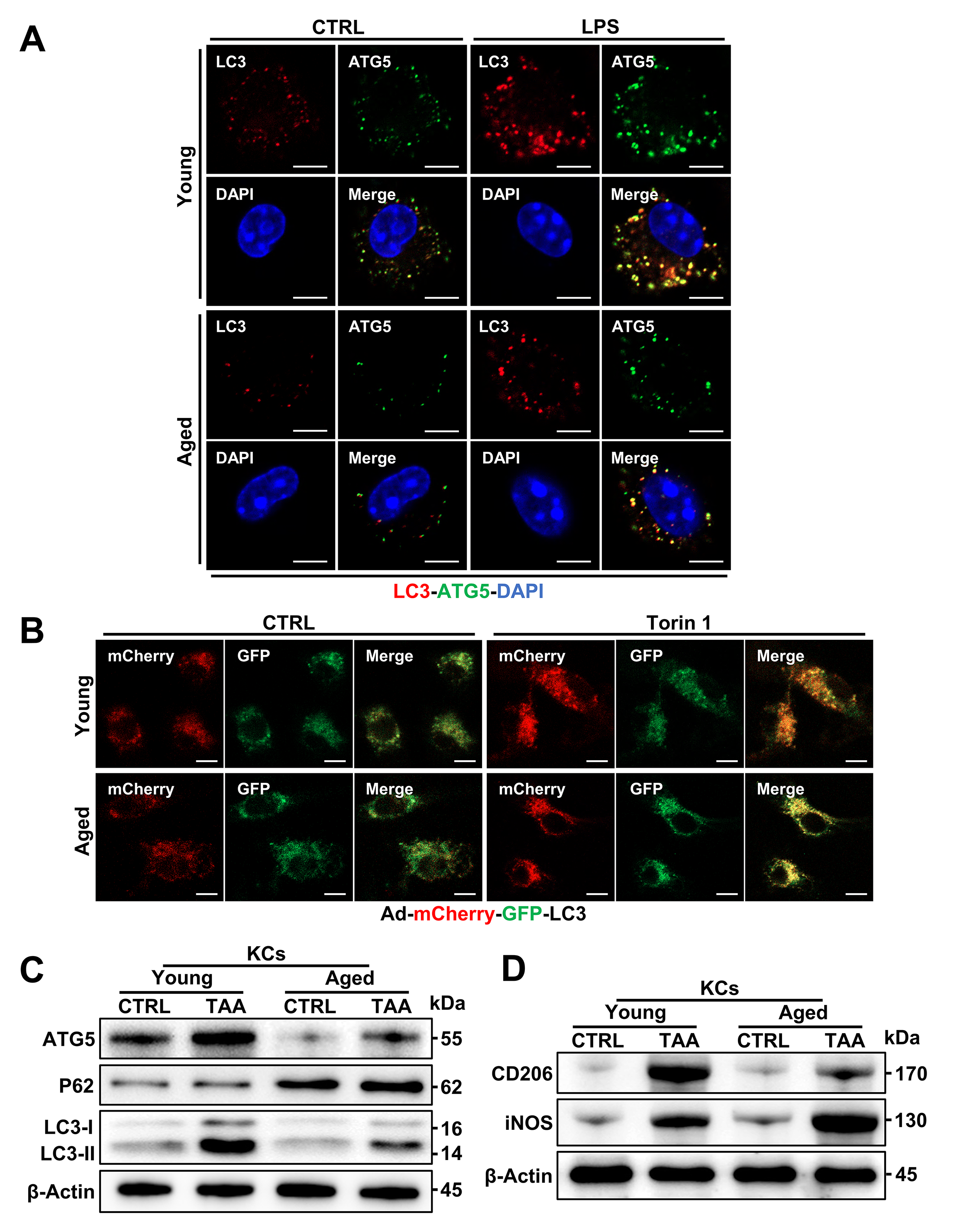
**

**Figure S1. Additional data on the effects of aging on autophagy and polarization of macrophages.** (A) Young and aged BMDMs derived from mouse bone marrow were infected with Ad-mCherry-GFP-LC3 (20 MOI) on the fifth day when the cells were not fully mature and then treated with Torin 1 (1 µM) at the seventh day. Representative laser confocal microscope images. (B) Young and aged BMDMs were then treated by LPS (100 ng/ml). Representative laser confocal microscope images of each group. (C-D) Kupffer cells (KCs) were isolated from young and aged mice with or without TAA treatment. Immunoblotting of LC3-I/II, ATG5, p62, CD206 and iNOS expression in BMDMs of each group.

**Supplementary Figure 2.**


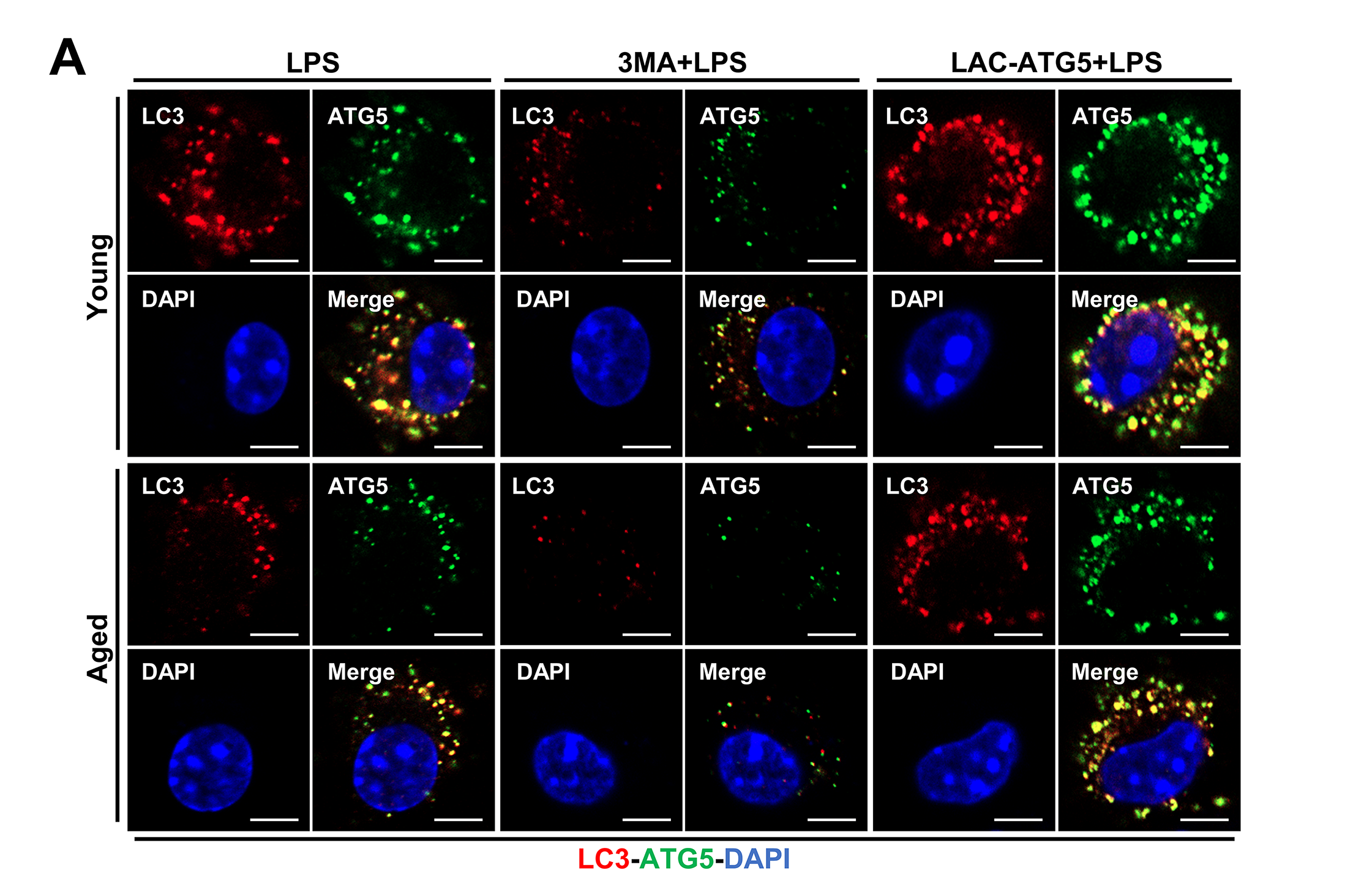


**Figure S2. Additional data of LC3 puncta of each cell.** (A) Young and aged BMDMs were treated with LPS (100 ng/ml) with or without pretreatment with 3-MA (5 mM) or LAC-ATG5 transfection. Representative laser confocal microscope images of each cell.

**Supplementary Figure 3.**

**
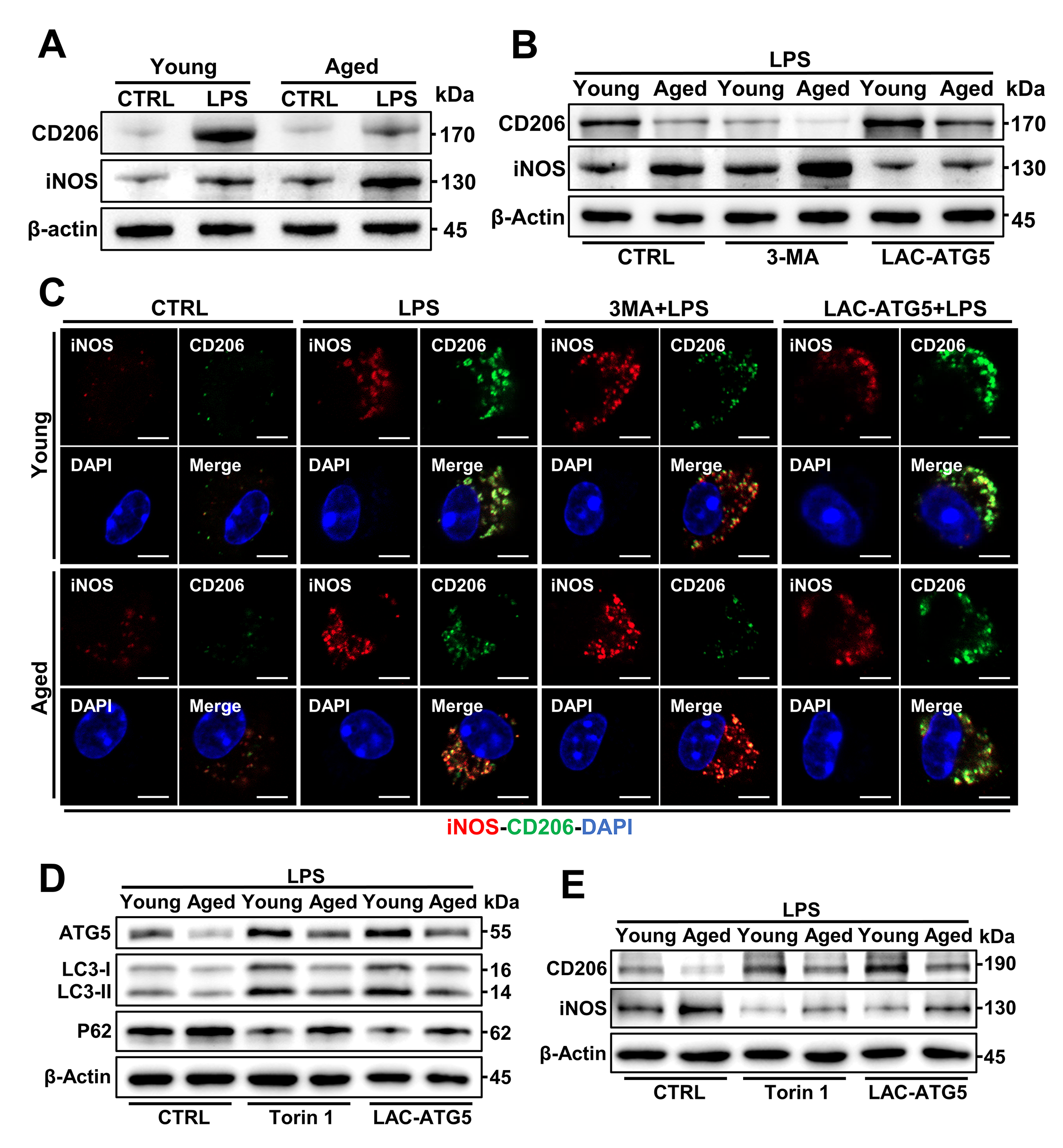
**

**Figure S3. Additional data on the effects of aging on macrophage polarization markers.** (A) Young and aged BMDMs were treated by LPS (100 ng/ml). Immunoblotting of CD206 and iNOS. (B) Then young and aged BMDMs were treated by LPS (100 ng/ml), with or without the pretreatment of 3-MA (5mM) or LAC-ATG5. Immunoblotting of CD206 and iNOS. (C) Representative laser confocal microscope images of each experimental group. (D-E) Young and aged BMDMs were treated by LPS (100 ng/ml), with or without the pretreatment of Torin 1 or LAC-ATG5. Immunoblotting of each experimental group.

**Supplementary Figure 4.**


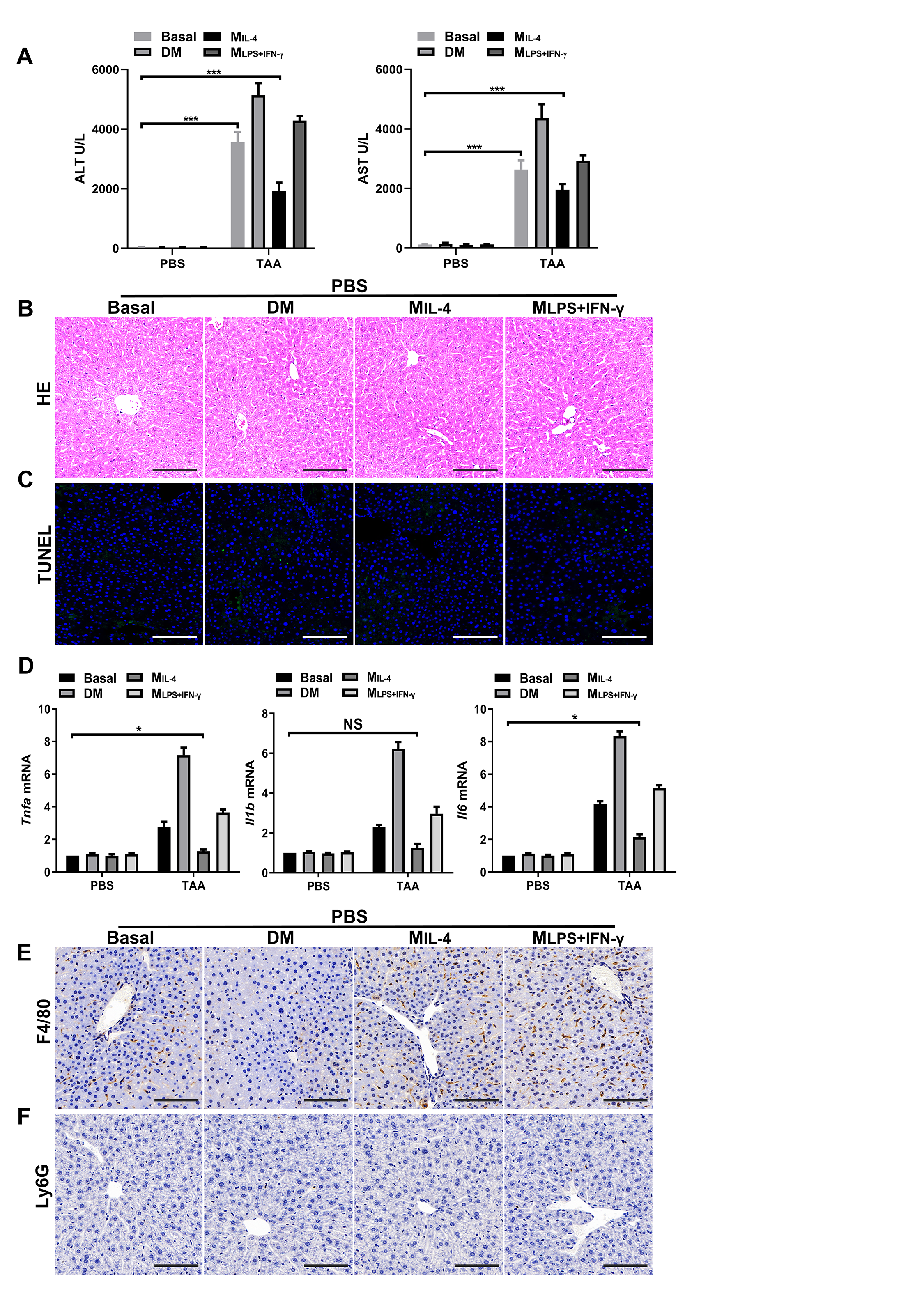


**Figure S4. The control group in the results of Figure 7.** We performed the following experimental groups: macrophage depletion by clodronate liposome injection (DM), transplantation of LPS+IFN-γ-induced BMDMs (M_LPS+IFN-γ_), and transplantation of IL-4-induced BMDMs (M_IL-4_). Results of TAA-ALI groups were shown in Figure 7, and the control (PBS treated) groups were shown here. (A) Representative H&E staining of liver. (B) Representative TUNEL (green fluorescence) staining of liver with DAPI counterstain (blue fluorescence). (C) Serum ALT (left panel) and AST (right panel) in each group. (D) mRNA expression (*Tnfa, Il1b, Il6*) of live tissue was detected by RT-PCR. The average target gene/GAPDH ratio of different experimental groups relative to the control group was given. (E) Representative F4/80 and (F) Ly6G IHC staining images of liver tissue from each group. All data shown were n = 6–10 mice per group. All results were representative of at least two independent experiments. Values were presented as the mean ± SD. *p ≤ 0.05; **p ≤ 0.01; ***p ≤ 0.001.

**Supplementary Figure 5.**


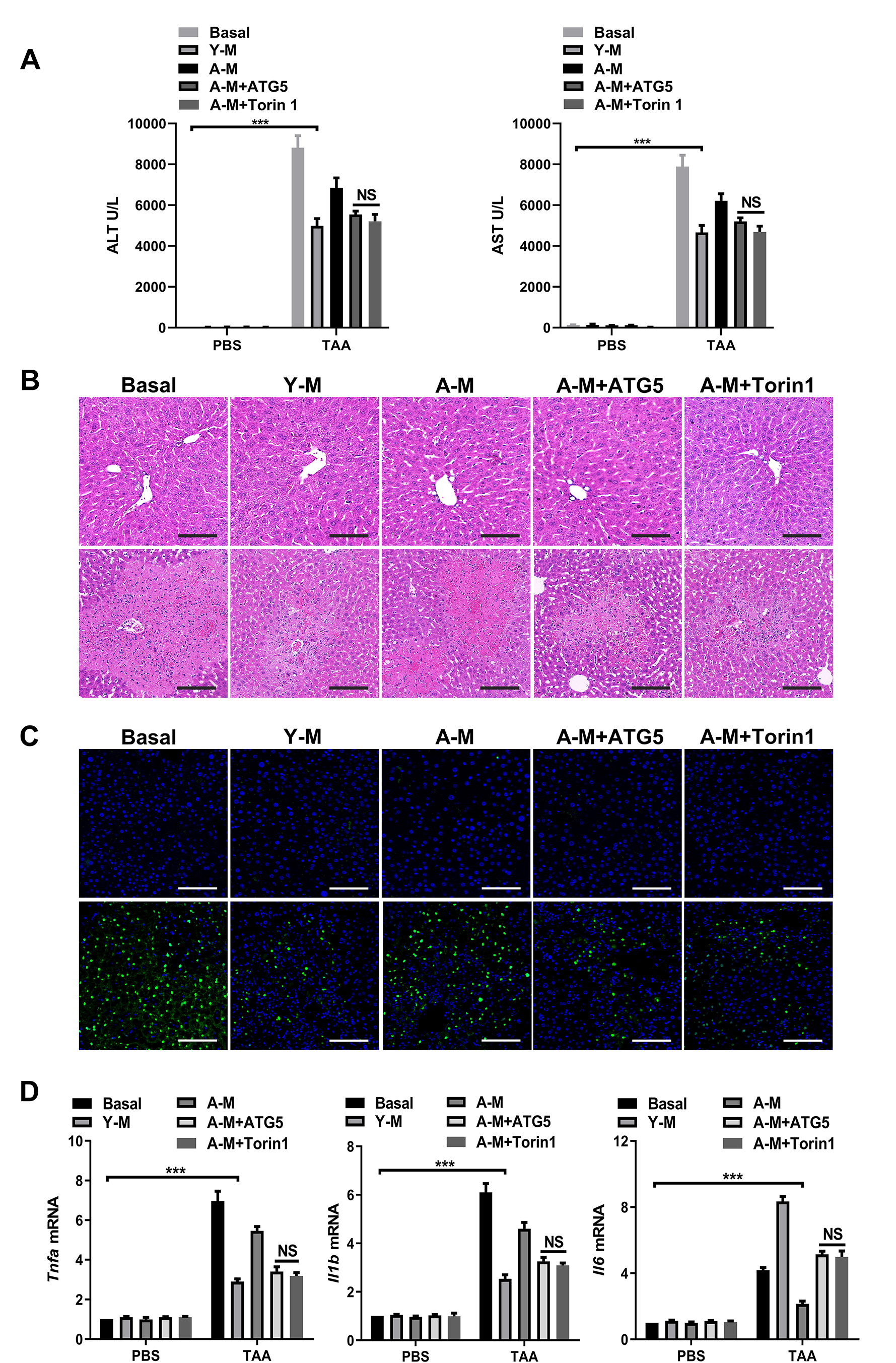


**Figure S5. Activation of autophagy by Torin 1 in macrophages can achieve liver injury protection similar to ATG5 rescue.** Young BMDMs (Y-M) and aged BMDMs (A-M) were isolated and derived from young and aged mice. Four macrophage populations, derived from mouse BM, were prepared: Y-M, A-M, and A-M with LAC-ATG5 (A-M+ATG5), A-M with Torin 1 pretreatment (A-M+Torin 1). After transplanting these four kinds of BMDMs by tail vein injection and control, TAA-ALI model was established respectively. (A) Serum ALT (left panel) and AST (right panel) in each group. (B) Representative H&E staining of liver. (C) Representative TUNEL (green fluorescence) staining of liver with DAPI counterstain (blue fluorescence). (D) mRNA expression (*Tnfa, Il1b, Il6*) of live tissue was detected by RT-PCR. The average target gene/GAPDH ratio of different experimental groups relative to the control group was given. All data shown were n = 6-10 mice per group. All results were representative of at least two independent experiments. Values were presented as the mean ± SD. *p ≤ 0.05; **p ≤ 0.01; ***p ≤ 0.001.

**Supplementary Table 1.**

**Table S1.** Primer sequences used for real-time RT-PCR

| *Gene* | *Forward/Reverse* | *Primer (5’ to 3’)* |
| --- | --- | --- |
| Mouse |  |  |
| *Gapdh* | Forward | ATGTTCCAGTATGACTCCACTCACG |
|  | Reverse | GAAGACACCAGTAGACTCCACGACA |
| *Il1b* | Forward  Reverse | GAAATGCCACCTTTTGACAGTG TGGATGCTCTCATCAGGACAG |
| *Il6* | Forward  Reverse | CTGCAAGAGACTTCC ATCCAG AGTGGTATAGACAGGTCTGTTGG |
| *Tnfa* | Forward  Reverse | CCTGTAGCCCACGTCGTAG GGGAGTAGACAAGGTACAACCC |
| *Il10* | Forward  Reverse | GCTCTTACTGACTGGCATGAG CGCAGCTCTAGGAGCATGTG |
| *Mrc1* | Forward  Reverse | CCACGGATGACCTGTGCTCGAG  ACACCAGAGCCATCCGTCCGA |
| *Mcp1* | Forward  Reverse | TCATGCTTCTGGGCCTGCTG TCTCATTTGGTTCCGATCCAGGTT |
| *iNOS* | Forward | CCAAGCCCTCACCTACTTCC |
|  | Reverse | CTCTGAGGGCTGACACAAGG |
| *Arg1* | Forward | CTCCAAGCCAAAGTCCTTAGAG |
|  | Reverse | AGGAGCTGTCATTAGGGACATC |
